# GABA Shunt in Durum Wheat

**DOI:** 10.3389/fpls.2018.00100

**Published:** 2018-02-02

**Authors:** Petronia Carillo

**Affiliations:** Dipartimento di Scienze e Tecnologie Ambientali, Biologiche e Farmaceutiche, Università degli Studi della Campania “Luigi Vanvitelli”, Caserta, Italy

**Keywords:** GABA, osmotic adjustment, glycine betaine, proline, combined stresses, daily light integral (DLI), salinity

## Abstract

Plant responses to salinity are complex, especially when combined with other stresses, and involve many changes in gene expression and metabolic fluxes. Until now, plant stress studies have been mainly dealt only with a single stress approach. However, plants exposed to multiple stresses at the same time, a combinatorial approach reflecting real-world scenarios, show tailored responses completely different from the response to the individual stresses, due to the stress-related plasticity of plant genome and to specific metabolic modifications. In this view, recently it has been found that γ-aminobutyric acid (GABA) but not glycine betaine (GB) is accumulated in durum wheat plants under salinity only when it is combined with high nitrate and high light. In these conditions, plants show lower reactive oxygen species levels and higher photosynthetic efficiency than plants under salinity at low light. This is certainly relevant because the most of drought or salinity studies performed on cereal seedlings have been done in growth chambers under controlled culture conditions and artificial lighting set at low light. However, it is very difficult to interpret these data. To unravel the reason of GABA accumulation and its possible mode of action, in this review, all possible roles for GABA shunt under stress are considered, and an additional mechanism of action triggered by salinity and high light suggested.

## Introduction

Salinity exerts pleiotropic effects which reduce plant growth, development, and survival by a multitude of mechanisms including alterations in water relations within the plant (Munns and Tester, 2008; Deinlein et al., 2014), ion deficiencies or toxicities (Tavakkoli et al., 2011), and oxidative stress (Munns and Tester, 2008; Gorham et al., 2010; Tavakkoli et al., 2011; AbdElgawad et al., 2016; Annunziata et al., 2017). The initial exposure of salt sensitive plants to salinity causes water stress, since plant root access to soil water is reduced by the increased osmotic strength of the soil solution (Carillo et al., 2011a). Osmotic stress changes cell water relations, causes inhibition of cell expansion and division and decreases stomatal aperture and transpiration (Negrão et al., 2017). During long-term exposure to salinity, plants undergo ionic stress, in particular due to sodium chloride, which affects protein synthesis, enzyme activities, and photosynthesis causing premature senescence of older leaves and chlorosis and necrosis of mature leaves (Hasegawa et al., 2000; Munns, 2002; Munns and Tester, 2008). Excess sodium is particularly harmful for plant cells because it substitutes potassium in key enzymatic reactions leading to enzyme inhibition, alteration of metabolic processes, plant nutritional imbalance, and oxidative stress. All these effects synergistically contribute to reduce plant growth, development, and survival. The metabolic perturbation in plants exposed to salinity involves a broad spectrum of metabolic pathways and both primary and secondary metabolism (Carillo et al., 2008, 2011a; Fraire-Velázquez and Balderas-Hernández, 2013). One of the mechanisms used by plants to minimize stress damages and re-establish growth involves toxic ions sequestration (in particular sodium and chloride) in the vacuole, as inexpensive osmotica, and production and accumulation of organic osmolytes in the cytosol for rapid osmotic adjustment and toxicity prevention (Annunziata et al., 2017). However, this ubiquitous and coordinated synthesis of protective metabolites, which principally are nitrogen containing molecules such as amino acids, amines, and betaines (Mansour, 2000), has a very high cost in terms of energy consumption (50–70 moles ATP for mole) (Raven, 1985; Cuin et al., 2009; Shabala, 2013). In addition to proline, glycine betaine (GB) is one of the main osmolytes found in Poaceae under salinity (Carillo et al., 2008). However, recent research on durum wheat shows that the complex interplay seen in plants under salinity at low light is different at high light with GB playing no role in it. On the contrary, the synthesis of γ-aminobutyric acid (GABA) in particular, and in minor way of other amino acids including proline, remodeled metabolism and defense processes, playing a key role in the response to simultaneous stresses (Woodrow et al., 2017). This result is worthy of attention because most of the studies done on cereal seedlings under salinity have been performed in controlled environment chambers operated with low-medium light levels up to about 300–350 μmol m^-2^ s^-1^ photosynthetic active radiation (PAR) corresponding to a daily light integral (DLI) of about 17–20 mol m^-2^ d^-1^ with a 16-h photoperiod (Annunziata et al., 2017). These light intensities correspond to the minimum light required to obtain the maximum efficiency of photosynthesis and maximum growth of seedlings (Bolton and Hall, 1991). Durum wheat plants growing in natural environments, on the contrary, can experience, in a clear day, light intensities rising up to 900 and 2000 μmol m^-2^ s^-1^ in winter and summer, respectively, which exceed their photosynthetic capacity (Carillo et al., 2011b).

## The Nature of Gaba Accumulation is Still a Controversial Issue

GABA is a non-protein amino acid that was first discovered in plants, being highly accumulated in response to biotic and abiotic stresses included senescence, and then in animal mature brain, where it plays a major role as an inhibitory neurotransmitter (Michaeli and Fromm, 2015). However, GABA can also function as trophic factor influencing nervous system differentiation, synapse maturation, and cell death (Owens and Kriegstein, 2002). Recently, it has been hypothesizes that GABA_A_ receptor signaling can function in animals for neuronal network synchronization both during development and in the adult brain (Avoli and Krnjević, 2016). GABA is a significant component of the free amino acids pool in bacteria, fungi, plants and animals, and in all these diverse organisms the enzymes involved in its metabolism are conserved (Shelp et al., 1999; Michaeli and Fromm, 2015).

GABA increase in plants has been found as a common response in concomitance with restriction of glutamine synthesis, reduction of protein synthesis and an increase in protein degradation (Bouché and Fromm, 2004). Under several stresses, GABA concentration can exceed that of amino acids involved in protein synthesis (Kinnersley and Turano, 2000). Therefore, many speculative functions in stress abiotic mitigation have been proposed. However, its function in plants is still a matter of debate and the reasons for its accumulation difficult to interpret (Fait et al., 2008; Renault et al., 2013; Michaeli and Fromm, 2015). A GABA shunt would supply NADH and/or succinate to tricarboxylic acid (TCA) cycle (Bouché and Fromm, 2004; Studart-Guimarães et al., 2007). In fact, Shelp et al. (2012) found that GABA transaminase (4-aminobutyrate:2-oxoglutarate aminotransferase, GABA-T) activity would be limited under stress conditions, determining an accumulation of GABA useful for the provision of anaplerotic succinate for the Krebs cycle upon stress relief, instrumental to cell viability. Accordingly, GABA-T deficient mutants *her1* and *gaba-t/pop2-1* underwent inhibition of root elongation, hypocotyl developmental defects and alterations of cell wall composition when there was no GABA-derived succinate in roots after removal of salt stress (Mirabella et al., 2008; Renault et al., 2013). However, GABA itself is also required to guide pollen tube growth to the ovary, stem elongation, ethylene emission, and gene induction (Kathiresan et al., 1998; Palanivelu et al., 2003; Mirabella et al., 2008; Renault et al., 2011). GABA has been associated with various physiological responses including nitrogen starvation (Recht et al., 2014) and sensing of nitrogen status (Lancien and Roberts, 2006; Mazzucotelli et al., 2006). In fact, GABA, together with calcium, is involved in 14-3-3-genes regulation, and since several key enzymes of nitrogen and carbon metabolism are 14-3-3 targets (e.g., nitrate reductase, glutamine synthetase, starch synthase III, and glyceraldehyde-3-P DH) it can be relevant in C–N balance (Lancien and Roberts, 2006; Roberts, 2007; Fait et al., 2011). Moreover, it can upregulate uptake of nitrate by mean of long-distance signaling pathway in *Brassica napus* (Beuve et al., 2004). GABA seems also involved in immune response in Arabidopsis since it can repress pathogenesis genes and lower *P. syringae* growth (Vest, 2013). It has a role as a component of the E-2-hexenal signaling pathway in response to biotic stresses (Mirabella et al., 2008); and given its role in neurotransmission, GABA could be synthetized also to discourage insect feeding delaying their development (Scholz et al., 2017). Therefore, in addition to its important role in providing precursors for anaplerosis of the TCA cycle, GABA can act as signaling molecule in plant growth and development (Bouché et al., 2004; Fait et al., 2008). Nevertheless, until recently the existence of receptors for this molecule has remained speculative (Molina-Rueda et al., 2015), and only in 2015 an aluminum-activated malate transporter (ALMT) channel activity was found, which determined changes in plant growth, suggesting ALMT proteins as ‘plant GABA receptor’ candidates (Ramesh et al., 2015; Gilliham and Tyerman, 2016). ALMTs are inhibited by GABA, and activated by malate and may serve as GABA receptors to regulate membrane potential by hyperpolarizing membranes and decreasing excitability (Hedrich et al., 2016).

## Gaba Shunt vs. Stress

Three enzymatic reactions are involved in the plant GABA metabolic pathway, called the GABA shunt because it bypasses two steps of the TCA cycle (Bouché and Fromm, 2004). It is mainly synthetized through a decarboxylation of glutamate catalyzed by glutamate decarboxylase (GAD, EC 4.1.1.15), dependent on pyridoxal phosphate. GABA is subsequently transported into the mitochondrion where it is catabolized by GABA transaminase (GABA-T, EC 2.6.1.19) to succinic semialdehyde (SSA). SSA can be oxidized by the mitochondrial succinic semialdehyde dehydrogenase (SSADH, EC 1.2.1.16) to produce succinate or reduced to γ-hydroxybutyrate (GHB) by the cytosolic γ-hydroxybutyrate dehydrogenase (GHBDH, EC 1.1.1.61) (Renault et al., 2010 and references therein). Under stress GAD activity undergoes changes of catalytic properties mediated either by calcium/calmodulin in rapid responses (Bouché et al., 2004), or by pH when cytosolic acidification occurs (Allan et al., 2009). Since the deficiency of GAD determines the presence of necrotic regions under standard light conditions and ROS accumulation in Arabidopsis, it was suggested that GABA level could be mainly controlled by the rate of its synthesis (Bouché et al., 2003; Fait et al., 2005). However, in Arabidopsis GABA-T deficient mutants *pop2-1*, GABA levels increased upon NaCl treatment proving that its concentration could also result from the rate of its degradation (Renault et al., 2010). GABA increase under salt stress could be also dependent on the reverse activity of GABA-T which can catalyze the backward reaction from SSA to GABA (Busch and Fromm, 1999; Akçay et al., 2012).

The GABA accumulated in the cytosol is then transferred to the mitochondria, via a mitochondrial GABA permease named GABP (Michaeli et al., 2011), and converted first to SSA by GABA-T and then to SSADH, which enters the TCA cycle (Bouché and Fromm, 2004). This latter step strongly affects the redox status of the cell because succinate bypasses three TCA cycle sites of NADH production (Fait et al., 2008). GABA-T and SSADH activities are probably coordinated in order to prevent SSA accumulation, and their coding genes (i.e., *POP2* and *SSADH*) are upregulated in situations in which GABA increases for example under moderate and high salinity (Renault et al., 2010). In fact, a correct functioning of the GABA shunt seems to be necessary to restrict reactive oxygen species (ROS) increase. *GABA-T* mutants are oversensitive to salinity (Renault et al., 2010), and *SSADH* knockout mutants show necrotic cell death caused by an abnormal accumulation of ROS, dwarfism, and hypersensitivity to environmental stresses (Fait et al., 2008).

It was shown that various abiotic as well as biotic stress stimuli induce an elevation of GABA level in plant tissue (Shelp et al., 1999; Kinnersley and Turano, 2000; Scholz et al., 2017). For example, it was observed that salt and cold stress or tissue damage of soybean (*Glycine max*) leaves lead to rapid accumulation of GABA up to 25-fold (Wallace et al., 1984; Ramputh and Bown, 1996). *Arabidopsis thaliana* plants producing a lower constitutive level of GABA showed a higher susceptibility to drought stress due to a stomata closure defect which could be rescued by increasing the internal GABA level (Mekonnen et al., 2016). Besides, external application of GABA to *Oryza sativa* seedlings, *Piper nigrum* and creeping bentgrass could enhance the individuals’ performance under heat and drought stress conditions, respectively (Nayyar et al., 2014; Li et al., 2016; Vijayakumari and Puthur, 2016). Abiotic stresses such as high light, heat, or UV favor the GABA shunt and the accumulation of GABA, too (Ludewig et al., 2008). In particular, NaCl salinity acts as a strong effector increasing GABA content (Xing et al., 2007; Allan et al., 2008; Bor et al., 2009; Renault et al., 2010, 2013; Akçay et al., 2012; Wang et al., 2017). Only Zhang et al. (2011) reported a GABA decrease in tobacco plants treated with 500 mM NaCl. This was probably due to the wrong stepwise increase of external NaCl applied to tobacco: plants passed from the 50 mM NaCl treatment of the first day to the 500 mM NaCl treatment of the second day, probably causing salt shock. On the contrary, the other studies in which GABA increased under salinity had gradual increases in NaCl treatments. Since salts in general, and NaCl in particular, increase slowly in soils, salt shock does not happen often in agricultural soils or natural habitats, except when there are coastal inundations (Carillo et al., 2011b; Woodrow et al., 2017).

## Gaba Accumulation in Durum Wheat Under Salinity is Nitrogen and Light Intensity Dependent

*Triticum turgidum* subsp. durum is a staple crop that is economically and culturally important in many European and North African countries that border the Mediterranean, being used for production of pasta, cous-cous and related products, but relatively neglected in agricultural research. It is often grown in marginal soils that are unsuitable for bread wheat (*Triticum aestivum*) cultivation, where plants are frequently exposed to environmental stresses, including drought, high temperatures, and high irradiance. Though, durum wheat, is more sensitive to salinity than bread wheat (James et al., 2006) and yields poorly on saline soil (Munns et al., 2006), due to a poor ability to exclude sodium from the leaf blades and a lack of the potassium–sodium discrimination character displayed by bread wheat (Gorham et al., 1990; James et al., 2006). Moreover, it is particularly vulnerable to salt stress at seedling stage (Gorham et al., 1990; James et al., 2006; Carillo et al., 2008).

As mentioned above, in durum wheat the main protective compounds against salt stress accumulated in leaves at low light are proline and GB (Carillo et al., 2005). These two osmolytes being exclusively present in the cytoplasm, which occupies less than 10% of cell volume, can significantly increase the osmotic pressure balancing the vacuolar osmotic potential (Cuin et al., 2009). Carillo et al. (2008) showed a spatial discrepancy in the synthesis of the two osmolytes in durum wheat under salinity: proline accumulates predominantly in older/senescing leaves when plants are grown with high nitrate fertilization, while GB preferentially accumulates in younger leaves independently of nitrogen nutrition. In addition to the spatial discrepancy, temporally the biosynthesis of these compounds is also asynchronous: proline tends to accumulate early, at the onset of the stress, while GB accumulates during prolonged stress (Carillo et al., 2008, 2011b).

**FIGURE 1:**
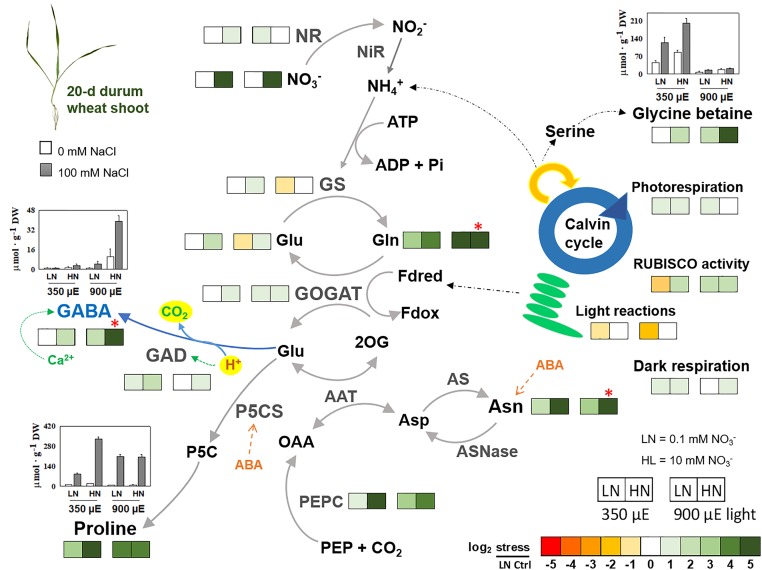
Heat map summarizing the effect of 100 mM NaCl salinity on nitrogen containing metabolites, enzyme activities, and gas exchanges in shoots of durum wheat seedlings grown in hydroponics with nutrient solutions supplemented with 0.1 or 10 mM KNO_3_, under low or high light intensity (350 or 900 μmol m^-2^ s^-1^ PAR, respectively). The metabolites considered are asparagine, glutamate, glutamine, GABA, proline, and glycine betaine. The enzymes are nitrate reductase (NR), glutamine synthetase (GS), glutamate synthase (GOGAT), GABA decarboxylase (GAD), phospho*enol*pyruvate carboxylase (PEPC) and RUBISCO. Results were calculated as Logarithm base 2 (log_2_) of salt stressed/control values and visualized using a false color scale, with green indicating an increase and red a decrease of values. Metabolites and enzymes are evaluated on a dry-weight basis: metabolites as μmol g^-1^ DW and enzyme activities as μmol h^-1^ g^-1^ DW. The values are mean ± SD (*n* = 4) (data from Carillo et al., 2005; Woodrow et al., 2017).

Therefore, nitrogen availability and use for the synthesis of these compatible compounds is crucial for plant stress survival (Krishna Rao and Gnanam, 1990; Silveira et al., 2001). Carillo et al. (2005, 2008, 2011b) have studied the effect of NaCl salinity on nitrogen metabolism of durum wheat seedlings grown in hydroponics under several different controlled conditions: low or high nitrate (0.1 and 10 mM KNO_3_, respectively) and low or high light intensity (350 or 900 μmol m^-2^ s^-1^ PAR, respectively). A heat map summarizing the experiments is shown in **Figure 1**. It considers amino acids like asparagine, glutamate, glutamine, proline, GABA and also the quaternary amine GB. This latter is accumulated only in plant at low light independently of nitrogen nutrition, with higher concentrations under salinity (Carillo et al., 2005, 2008). **Figure 1** shows an increase in proline in response to NaCl under low nitrate conditions also, though to a lesser extent than under high N conditions. Probably, glutamate was partially used as precursor of proline under low nitrate and high light; while, under high nitrate and high light, it was used to synthetize mainly GABA, that strongly accumulated in a calcium and pH dependent-manner (Woodrow et al., 2017). In addition, Carillo colleagues studied in the same plants the activity of enzymes involved in nitrogen metabolism, specifically nitrate reductase (NR), glutamate synthase (GOGAT), glutamine synthetase (GS), glutamate dehydrogenase (GDH), GAD and phospho*enol*piruvate carboxylase (PEPc). The enzyme activities appeared positively correlated to the compatible compounds content in leaves under salinity. In particular in young tissues at low light and independently of nitrate, GS, GOGAT, PEPC participated in the recycling of photorespiratory ammonia leading to the synthesis of GB in low nitrate plants (Carillo et al., 2008). At high nitrate, NR, GS, GOGAT, and PEPcase contributed to the nitrogen re-assimilation and *de novo* synthesis of amino acids, and in particular of proline and asparagine independently of light intensity, and of GABA thanks also to the concomitant action of GAD, but only at high light (Woodrow et al., 2017).

**FIGURE 2:**
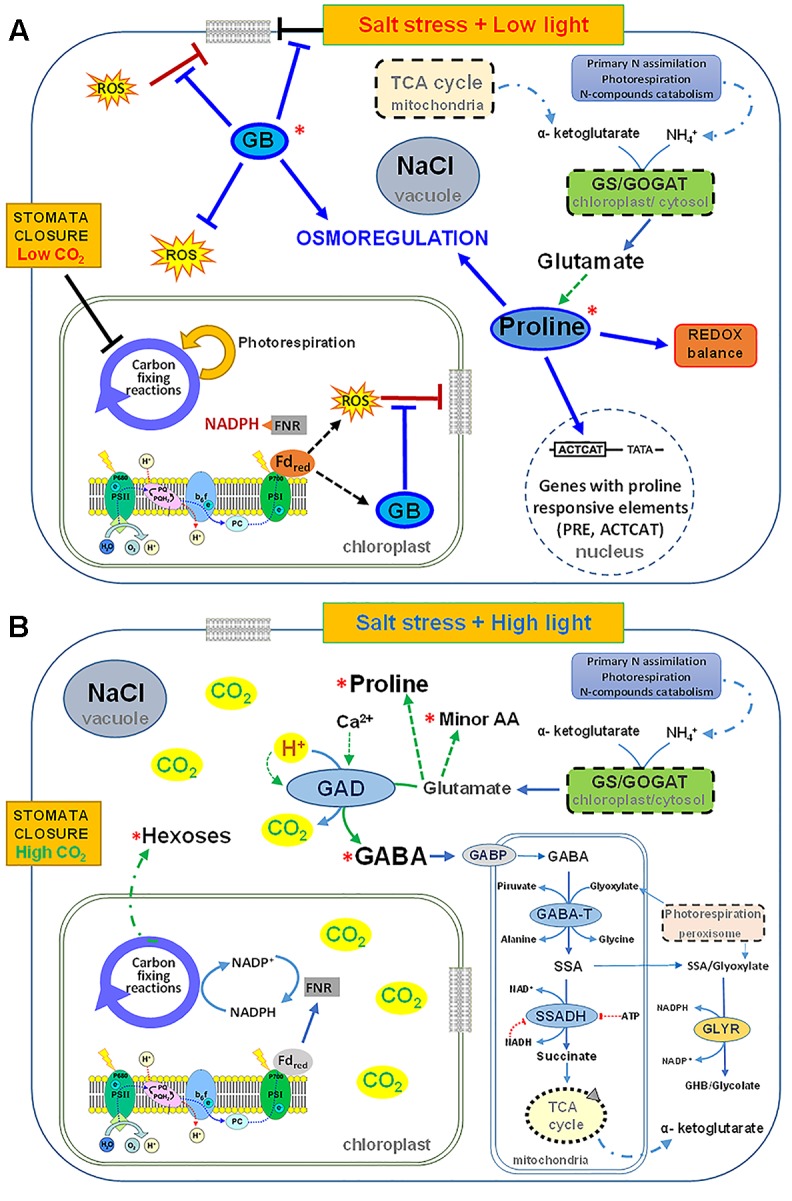
Scheme of the possible roles exerted by glycine betaine (GB) and proline under salinity as single stress **(A)** and by GABA under salinity and high light **(B)** in shoots of durum wheat plants grown in hydroponic culture supplemented with KNO_3_ 10 mM.

Proline is the most widely distributed osmolyte in plants, even if the nature of its accumulation under stress is still debated. It is not yet clear if it is a symptom of stress, a stress response or an adaptative strategy. However, proline can have many functions: in addition to its supposed role as osmolyte, it is able to stabilize membranes and proteins, scavenge ROS, buffer cellular redox potential, induce expression of salt stress responsive genes, in particular genes with proline responsive elements (e.g., PRE, ACTCAT) in their promoters and proline can be rapidly metabolized when no longer required (Woodrow et al., 2017) (**Figure 2A**). On the contrary, GB is present in several halophytes but only in few crop plants (in particular in Poaceae). It is an amphoteric compound, electrically neutral across a broad range of pH values and highly soluble in water. It can act as osmoregulator, interact and stabilize structure and activity of macromolecules, maintain the integrity of membranes against stresses, and scavenge ROS (Gupta and Huang, 2014). GB is synthesized after prolonged stress, especially in young tissues even at low nitrogen nutrition, in chloroplasts from choline through a two-step oxidation of choline, catalyzed by choline monooxygenase (CMO) and betaine aldehyde dehydrogenase, respectively. Since, it cannot be metabolized, even if easily and efficiently transported in plant tissues (Carillo et al., 2008), it must have a very important role as compatible solute. It could protect against salt stress young leaves and root tissues. Probably, the damages found in root tips of durum wheat under salinity, in particular the arrest of growth and differentiation could be ascribed to the delay in the synthesis of GB.

When GB synthesis and accumulation takes place, GABA is not synthetized under salinity. GB, together with proline, is so efficient in protecting salt stressed plants not only from osmotic imbalance, but also from oxidative stress, that, when they are present, antioxidant metabolites and enzymes do not play a main role in plant protection (Carillo et al., 2011b; Annunziata et al., 2017).

However, notwithstanding the important role of GB for young tissues under salinity, it is not synthesized under high light even in presence of salinity. The reason is probably that under high light, and independently of salinity, durum wheat CMO transcript undergoes a 60% intron retention, suggesting that high light can repress intron splicing leading to unproductive transcripts (Woodrow et al., unpublished data). A high light induced intron retention has been previously reported in *Physcomitrella patens* for genes functioning in chloroplast (Wu et al., 2014). In absence of GB, high concentration of calcium and a decreasing pH, GABA synthesis is induced (Woodrow et al., 2017).

The same trend of fast synthesis of proline and delayed synthesis of GB (Carillo et al., 2008) was also found in greenhouse grown bread wheat (Colmer et al., 1995). However, even if plants were exposed to a midday PAR of about 1100–1200 μmol m^-2^ s^-1^, similar to that used by Carillo et al. (2011b) and Woodrow et al. (2017), that is 900 μmol m^-2^ s^-1^ PAR, they did not show the same GB synthesis inhibition. It is important to specify that in growth chambers plants are usually grown with a constant (square-wave) irradiance during the day, whereas sunlight (also in the greenhouse) changes in a sinusoidal manner, fluctuating because of cloud cover or shading, and peaking at noon without sudden transitions at dawn and dusk (Annunziata et al., 2017). However, differences in GB synthesis may be also due to the use of two different species *T. aestivum* and *T. durum*.

In durum wheat plants under high light condition, glutamate strongly decreases, too (Carillo et al., 2011b; Woodrow et al., 2017). Glutamate decrease has been related to the synthesis of proline and minor amino acids at low nitrate; however, at high nitrate, its decrease seems mainly the result of calcium-dependent decarboxylation for γ-aminobutyric acid (GABA) production (Woodrow et al., 2017). Indeed, a strong correlation between calcium and GABA was shown by Woodrow et al. (2017), according to the calcium/calmodulin control of the GAD cytosolic decarboxylation of glutamate to GABA (Snedden and Fromm, 2001). However, *TdGAD* transcripts did not increase while GABA did in durum wheat shoots under high light and salinity. Akama and Takaiwa (2007), on the contrary, found that rice plants over-expressing *OsGAD* gene were able to accumulate GABA at high concentration under short-term salinity. However, GAD transcript and activity were not correlated to GABA accumulation in *Nicotiana sylvestris* under long-term salinity.Therefore, increases in GABA accumulation under salt stress could be induced by the reverse activity of GABA transaminase(GABA-T), which catalyzes not only the degradation of GABA but also the backward reaction from SSA to GABA (Busch and Fromm, 1999; Akçay et al., 2012). Accordingly, Renault et al. (2010) found that GABA-T deficient Arabidopsis mutants are oversensitive to NaCl treatment.

GAD and/or GABA-T activity increase can lead to high GABA concentration, at the expense of glutamate pools in leaves and reproductive tissues (Baum et al., 1996), controlling glutamate homeostasis (Masclaux-Daubresse et al., 2002). GABA accumulated in high nitrate durum wheat shoots under combined salinity and high light could participate in retaining the viability and photosynthetic efficiency of the plants, contributing to the avoidance of the impairment of the antioxidant defense mechanisms of cereal plants and the increase of ROS with subsequent photo-damage (Noctor and Foyer, 1998; Miller et al., 2010).

The GABA accumulated in durum wheat plants at high light and high nitrate could serve as temporary nitrogen store to decrease the excess of ammonium accumulated under salt stress also due to protein and amino acid catabolism and/or photorespiration (Woodrow et al., 2017). Moreover, it could be part of a biochemical mechanism enabling the stationary control of pH, because for its synthesis protons are consumed buffering stress-induced cytosolic acidosis (Shelp et al., 1999; Kinnersley and Turano, 2000; Fait et al., 2008; Molina-Rueda et al., 2015; Woodrow et al., 2017). As zwitterion, it can accumulate in plant cells acting as osmolyte without toxic effects (Renault et al., 2010; Signorelli et al., 2015). Kinnersley and Turano (2000) found that GABA accumulated in the cytosol could balance the decrease in water potential during cellular dehydration.

Moreover, it can exhibit a scavenging activity against superoxide anion radicals, singlet oxygen, and hydrogen peroxide exceeding those of proline and GB at the same concentration for the stabilization and protection of thylakoids and macromolecules (Liu et al., 2011; Molina-Rueda et al., 2015). GABA and proline are accumulated together also in response to salinity and water stress (Li et al., 2016). They can be rapidly synthetized for cell protection against stress, mainly as osmolytes and ROS scavengers, and broken down upon relief of stress to provide energy, carbon and nitrogen to recover and repair stress induced damages (Hare and Cress, 1997; Carillo et al., 2008). Since glutamate is the precursor of both proline and GABA, the higher ROS scavenger activity of GABA could account for the preferential increased GABA synthesis over proline in durum wheat, as well as in tobacco leaves under water stress (Liu et al., 2011).

## Conclusion

In durum wheat plants under high salinity at low light, the salt induced stomata closure restricts CO_2_ exchange consequently reducing the enzymatic CO_2_-fixation activity of the Calvin cycle, while increasing the over-excitation of the photosynthetic apparatus and the production of ROS. In this condition, GB synthesis is induced in chloroplasts, for increasing the protection of photosynthetic apparatus (Chen and Murata, 2011; Kurepin et al., 2015). GB, together with proline, contributes to scavenge ROS, osmoregulate the cytosolic compartments, stabilize membranes, buffer redox potential and induce salt responsive genes (**Figure 2A**).

However, the GABA shunt may play a more important role under combined salinity and high light at high nitrate. It is here suggested that the synthesis of GABA by glutamate decarboxylation catalyzed by GAD could contribute to the dissipation of excess of energy and release CO_2_, allowing the Calvin cycle to function while exerting a lower pressure on photosynthetic electron chain and decreasing ROS and photo-damage. Moreover, GABA shunt can supply NADH and/or succinate to the mitochondrial electron-transport chain under conditions in which respiration and TCA cycle are impaired and ROS increased (Bouché et al., 2003) (**Figure 2B**).

This hypothesis underlines the plasticity of plants that are able to respond to combined stresses enacting tailored responses completely different from those shown under single stresses. Therefore, it is necessary for the future to propose a combinatorial approach reflecting real-world scenarios to provide fundamental biological knowledge about how plants respond to multifactorial abiotic stresses. By understanding responses to single and multiple stresses, it will identify which aspects are linked to stress tolerance in general, and to what extent the overall response requires compromises between conflicting responses to different stresses. Only by exploring the molecular mechanisms underlying the multifaced responses of plants to environment and the trade-offs, it will be possible to program targeted interventions to improve the tolerance of plant crops and in particular of cereals to single and multifactorial abiotic stress.

## Author Contributions

PC conceived and wrote the paper. The author confirms being the sole contributor of this work and approved it for publication.

## Conflict of Interest Statement

The author declares that the research was conducted in the absence of any commercial or financial relationships that could be construed as a potential conflict of interest.
